# Natriuretic Peptides as Predictors for the Diagnosis of Pulmonary Hypertension Secondary to Left Heart Disease and for the Assessment of Its Severity

**DOI:** 10.3390/ijms27177776

**Published:** 2026-08-30

**Authors:** Filip Sawczak, Agata Kukfisz, Aleksandra Soloch, Kamila Kurkiewicz-Sawczak, Magdalena Dudek, Ewa Straburzyńska-Migaj, Marta Kałużna-Oleksy

**Affiliations:** 1 College of Medical Sciences, SGMK Copernicus University, al. Pilsudskiego 11/17, 10-545 Olsztyn, Poland; fsawczak@gmail.com; 2 1st Department of Cardiology, Poznan University of Medical Sciences, Dluga 1/2, 61-848 Poznan, Poland; ola.soloch@gmail.com (A.S.);; 3 3rd Department of Cardiology, Silesian Center for Heart Diseases, Medical University of Silesia, 40-055 Zabrze, Poland; 4 Department of Paediatric Cardiology, Poznan University of Medical Sciences, Szpitalna 27/33, 60-572 Poznan, Poland; kkurkiewicz75@gmail.com

**Keywords:** heart failure with reduced ejection fraction, right heart catheterization, systolic dysfunction, biomarker, N-terminal pro-B-type natriuretic peptide, B-type natriuretic peptide, pulmonary vascular resistance, pulmonary artery pressure, cardiac index

## Abstract

Pulmonary hypertension (PH) frequently complicates heart failure with reduced ejection fraction (HFrEF) and worsens prognosis. Right heart catheterization (RHC) remains the diagnostic gold standard, but natriuretic peptides may help identify patients requiring invasive assessment. We retrospectively analyzed 563 HFrEF patients who underwent RHC. Patients with and without PH were compared. Associations between B-type natriuretic peptide (BNP), N-terminal pro-B-type natriuretic peptide (NT-proBNP) and RHC parameters were assessed using correlations, restricted cubic splines, logistic regression and receiver operating characteristic (ROC) curves. PH was present in 443 patients (78.7%). Of the study group, 87 (15.5%) were females and 476 (84.5%) were males, the median age was 55 years and the median ejection fraction was 20%. Patients with PH had significantly higher BNP (*p* < 0.001), NT-proBNP (*p* < 0.001), New York Heart Association (NYHA) class (*p* < 0.001) and lower ejection fraction (*p* < 0.001). Increase in PH risk (*p* < 0.001), mean pulmonary artery pressure (*p* < 0.001), pulmonary vascular resistance (PVR) (*p* < 0.001) and pulmonary arterial wedge pressure (*p* < 0.001) and decrease in cardiac index (*p* < 0.001) were associated with an increase in BNP up to approximately 500 pg/mL and NT-proBNP up to approximately 3000 pg/mL. BNP and NT-proBNP were independent predictors of PH and PVR > 5 Wood units. BNP and NT-proBNP predicted PH with area under the curve (AUC) 0.798 and 0.727, respectively, while prediction of PVR > 5 Wood units (AUC 0.661 and 0.664, respectively) or cardiac index < 2.0 L/min/m^2^ was less accurate (AUC 0.607 and 0.623, respectively). Measurement of plasma natriuretic peptides may support PH screening in HFrEF, but their limited ability to detect severe precapillary component confirms RHC as the definitive tool for hemodynamic phenotyping.

## 1. Introduction

Cardiovascular diseases are the most common cause of mortality worldwide [1]. Heart failure (HF) is a complex syndrome of symptoms associated with impaired physical performance, reduced quality of life and high mortality [2]. Despite advances in diagnosis and treatment, prognosis remains unfavorable [2]. Additionally, HF patients often develop pulmonary hypertension (PH), which further worsens the prognosis [3,4]. Left heart diseases (LHD) are the leading cause of PH [5]. Elevated left-sided filling pressure may be estimated by Doppler echocardiography. However, right heart catheterization (RHC), which enables assessment of pulmonary arterial wedge pressure (PAWP) and pulmonary vascular resistance (PVR), remains the “gold standard” to diagnose PH due to LHD (PH-LHD) [5]. Moreover, measurement of PVR is needed to diagnose a severe precapillary component, defined as PVR > 5 Wood units [5]. According to the 2022 European Society of Cardiology/European Respiratory Society (ESC/ERS) PH guidelines, this diagnosis should prompt an individualized therapeutic approach [5].

However, RHC is an invasive procedure with limited availability and may be associated with serious complications. Therefore, noninvasive tools that help identify patients most likely to benefit from RHC are needed. As blood tests are readily available and routinely performed, using a biomarker [6,7], ideally one already in clinical use, would be advantageous [8]. Optimally, selected biomarkers could be used to monitor treatment response in PH-LHD and may even help predict treatment success [9]. Natriuretic peptides, including B-type natriuretic peptide (BNP) and N-terminal pro-B-type natriuretic peptide (NT-proBNP), are among the most important biomarkers in the diagnostic and therapeutic management of HF patients [2]. They are associated with prognosis in HF [2,10], as well as in pulmonary arterial hypertension and chronic thromboembolic PH [5]. Natriuretic peptides are released by cardiomyocytes in response to increased myocardial wall stress caused by pressure or volume overload of both left and right ventricles [11]. The precursor propeptide synthesized in cardiomyocytes is subsequently cleaved into biologically active BNP and biologically inactive NT-proBNP. In theory, BNP and NT-proBNP should be secreted from cardiomyocytes in equimolar amounts. However, in clinical practice, the molar concentration of NT-proBNP in peripheral blood usually exceeds that of BNP [12,13,14,15,16]. BNP is removed from the circulation through binding to natriuretic peptide receptor type C (NPR-C) and by proteolytic degradation mediated by neprilysin and neprilysin 2 [15,17]. By contrast, NT-proBNP neither binds to NPR-type A (NPR-A) or NPR-C nor undergoes cleavage by neutral endopeptidases. Consequently, NT-proBNP has a longer plasma half-life than BNP (120 vs. 20 min) [15,18]. Moreover, renal function appears to have a greater impact on NT-proBNP elimination compared with BNP clearance [19]. In HF, NT-proBNP-directed therapy proved to be as effective as the conventional guidelines-directed medical therapy in randomized clinical trials in both acute and chronic HF [20,21]. Beyond heart failure, NT-proBNP also plays a central role in pulmonary arterial hypertension and chronic thromboembolic PH, where it is incorporated into risk stratification tools that guide therapeutic decisions [5,22,23,24,25]. NT-proBNP < 300 pg/mL or BNP < 50 pg/mL are considered a criterion of favorable prognosis in most algorithms [22,24,25] and are one of the goals of therapy [5]. However, it remains insufficiently established whether natriuretic peptides, in patients with HF with reduced ejection fraction (HFrEF) can reliably identify PH-LHD, and particularly a severe precapillary component. The authors of this study hypothesize that natriuretic peptides are associated with pulmonary hemodynamics in HFrEF and may help predict the presence of PH, allowing better selection of patients for RHC and potentially avoiding unnecessary RHC in some patients.

This study aimed to evaluate whether natriuretic peptides concentrations (BNP and NT-proBNP), could accurately predict pulmonary hypertension and PH with a severe precapillary component in patients with HFrEF. Additionally, we aimed to evaluate the relationships between natriuretic peptide concentrations and mean pulmonary artery pressure (PAP), PVR, PAWP, and cardiac index (CI), including their potential nonlinearity.

## 2. Results

### 2.1. Characteristics of Patients

**Figure 1:**
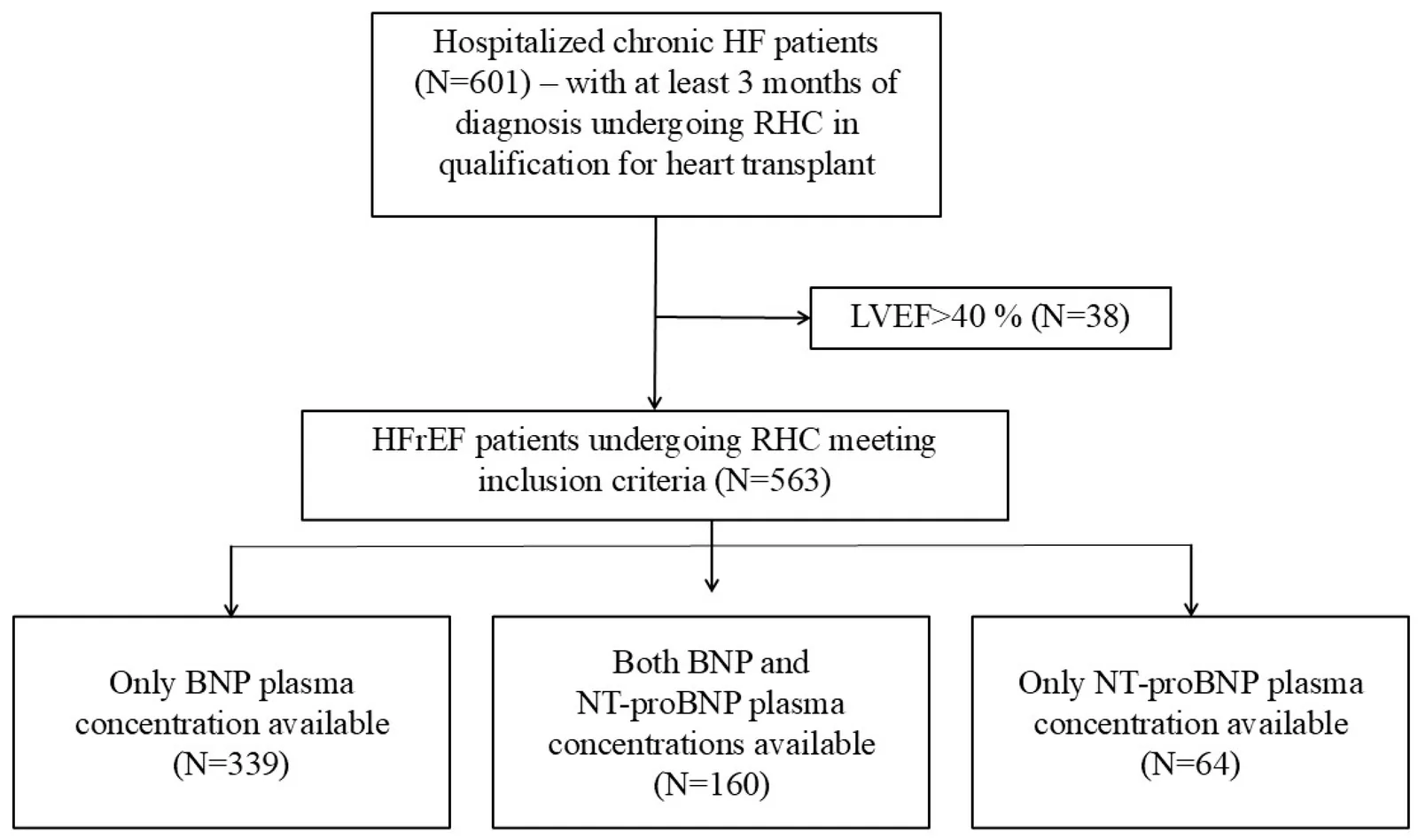
Flowchart visualizing enrollment process. BNP, B-type natriuretic peptide; HF, heart failure; LVEF, left ventricular ejection fraction; NT-proBNP, N-terminal pro-B-type natriuretic peptide; PH, pulmonary hypertension; RHC, right heart catheterization.

The study included 563 patients; 443 (78.7%) had PH (Figure 1). Median age was 55 years (IQR, 46–60) (Table 1, Supplementary Table S1). In total, 15.5% (87) of study participants were females and 84.5% (476) were males; 69.3% were in New York Heart Association (NYHA) class III or IV before the RHC procedure. Median left ventricular ejection fraction (LVEF) was 20% (IQR, 15–25). Baseline characteristics of the analyzed group are presented in Table 1 and Supplementary Table S1.

### 2.2. Comparison of Patients with and Without PH

Patients were divided into two groups according to the presence of PH, and both groups were compared (Table 1, Supplementary Table S1). The patients with PH did not differ significantly from those without PH in age or HF etiology. Females were less common in the group with PH (13.8% vs. 21.7%; *p* = 0.034). The PH patients had NYHA class III and IV significantly more often (73.8% vs. 52.5%; *p* < 0.001). The patients with PH had significantly higher BNP concentration [598.4 pg/mL (IQR, 323.0-1119.2) vs. 163.7 pg/mL (IQR, 83.2–408.7 pg/mL); *p* < 0.001], higher NT-proBNP [2729 pg/mL (IQR, 1595–5493) vs. 1089 pg/mL (IQR, 461–2632); *p* < 0.001], higher thyroid-stimulating hormone (TSH) [1.80 uIU/mL (IQR, 1.02–3.02) vs. 1.36 uIU/mL (IQR, 0.79–2.40); *p* < 0.001] and lower glomerular filtration rate (GFR) [68.0 mL/min/1.73 m^2^ (SD, 21.7) vs. 76.1 mL/min/1.73 m^2^ (SD, 25.4); *p* < 0.001]. Molar ratio of NT-proBNP/BNP was lower in patients with PH [2.17 (IQR, 1.30–2.62) vs. 2.97 (IQR, 2.27–3.79)]. Considering echocardiography, LVEF was lower in PH patients than in those without PH [20% (IQR, 15–25) vs. 25% (IQR, 20–26.5); *p* < 0.001]. Dimensions of the left atrium [54 mm (IQR, 49–59) vs. 49 mm (IQR, 43–54); *p* < 0.001], left ventricle [73.1 mm (SD, 11.3) vs. 70.6 mm (SD, 12.1); *p* = 0.041], and right ventricle (RV) [36 mm (IQR, 32–41) vs. 33 mm (IQR, 30–37); *p* < 0.001] were significantly higher in patients with PH. Moderate to severe mitral regurgitation was more common in the PH patients; it was present in 82.9% compared to 50.1% of the non-PH patients (*p* < 0.001). Similarly, moderate to severe tricuspid regurgitation was present in 44.4% of patients with pulmonary hypertension compared to the 18.1% in the non-PH group (*p* < 0.001). Median right ventricular systolic pressure assessed by echocardiography (RVSP) was 45 mmHg (IQR, 36–55) in patients with mean PAP > 20 mmHg and 34 mmHg (IQR, 30–38) in patients with normal pulmonary pressure assessed by RHC (*p* < 0.001). Correlations between RHC parameters and various clinical, biochemical and echocardiographic parameters were assessed. Natriuretic peptides concentrations were correlated with most RHC parameters, including mean PAP, PVR, PAWP, CI and aortic systolic and mean pressures (Supplementary Table S2).

### 2.3. Stratification by Natriuretic Peptides Concentrations

**Figure 2:**
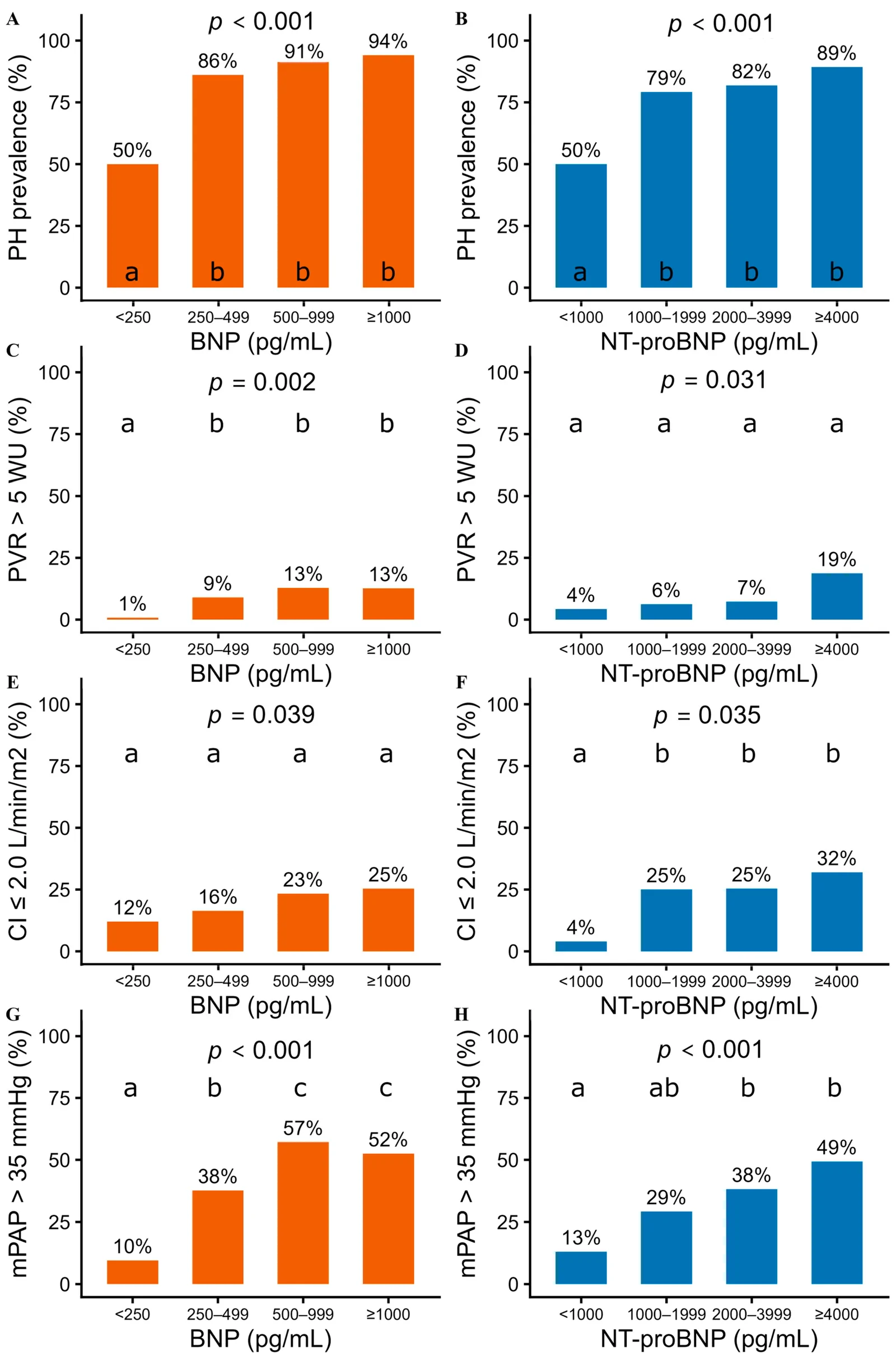
Prevalence of PH (**A**,**B**), and selected hemodynamic characteristics: PVR > 5 Wood units (**C**,**D**), CI ≤ 2.0 L/min/m^2^ (**E**,**F**), and mean PAP > 35 mmHg (**G**,**H**) across groups with different concentrations of BNP and NT-proBNP. *p*-values < 0.05 were considered significant. Groups not sharing a common letter (a,b,c,d) differed significantly in Holm-adjusted post hoc comparisons. The Cochran–Armitage trend test was calculated to assess presence of increasing trends across BNP and NT-proBNP strata (*p* for Cochran–Armitage trend test: A < 0.001; B < 0.001; C < 0.001; D 0.008; E 0.003; F 0.001; G < 0.001; H < 0.001). BNP, B-type natriuretic peptide; CI, cardiac index; mean PAP, mean pulmonary artery pressure; NT-proBNP, N-terminal pro-B-type natriuretic peptide; PH, pulmonary hypertension; PVR, pulmonary vascular resistance.

Furthermore, patients were divided according to the NT-proBNP and BNP concentrations. The selected cutoff values (250, 500, 1000 for BNP and 1000, 2000, 4000 for NT-proBNP) divided enrolled patients into four groups of similar size (Figure 2, Supplementary Table S3). In total, 50% of patients with BNP < 250 pg/mL or NT-proBNP < 1000 pg/mL had PH. The prevalence of PH increased with higher natriuretic peptides concentrations—up to 94% for BNP ≥ 1000 pg/mL and 89% for NT-proBNP ≥ 4000 pg/mL (*p* for trend < 0.001). Similarly, the prevalence of severe precapillary component defined as PVR > 5 Wood units increased with higher natriuretic peptides plasma concentrations, ranging from 1% for BNP < 250 pg/mL up to 13% for BNP ≥ 1000 pg/mL and 19% for NT-proBNP ≥ 4000 pg/mL (*p* for trend < 0.001 for BNP and 0.008 for NT-proBNP concentrations). The prevalence of low cardiac index, defined as CI ≤ 2.0 L/min/m^2^, also increased with higher natriuretic peptides concentrations, ranging from 4% for NT-proBNP < 1000 pg/mL up to 25% for BNP ≥ 1000 pg/mL and 32% for NT-proBNP ≥ 4000 pg/mL (*p* for trend 0.001 for BNP and 0.003 for NT-proBNP concentrations). Consistently, the prevalence of mean PAP > 35 mmHg increased with higher BNP and NT-proBNP plasma concentrations, ranging from 10% for BNP < 250 pg/mL up to about 50% of patients with BNP > 500 pg/mL or NT-proBNP ≥ 4000 pg/mL (*p* for trend < 0.001).

**Figure 3:**
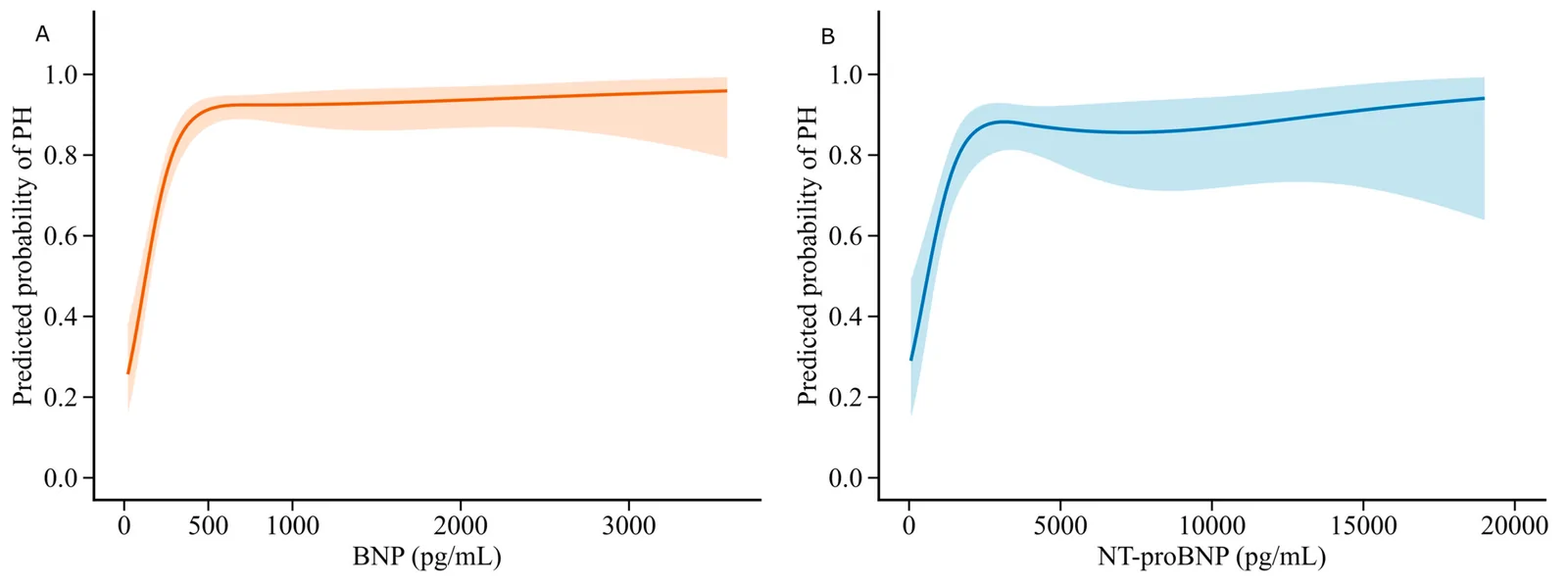
Restricted cubic spline demonstrating the nonlinear association between BNP (**A**), NT-proBNP (**B**) and the probability of pulmonary hypertension. The solid line represents the predicted values, and the shaded area indicates 95% confidence intervals. *p*-overall refers to the significance of the overall association, whereas *p*-nonlinearity assesses deviation from linearity. *p*-overall: A < 0.001; B < 0.001; *p*-nonlinearity: A < 0.001; B < 0.001. BNP, B-type natriuretic peptide; NT-proBNP, N-terminal pro-B-type natriuretic peptide.

The correlation between natriuretic peptides concentrations and PH was further assessed with restricted cubic splines predicting PH according to BNP and NT-proBNP concentrations (Figure 3). The risk of PH increased gradually until BNP concentration reached approximately 500 pg/mL, and about 3000 pg/mL of NT-proBNP and plateaued thereafter at a high level (>80%) (*p* for nonlinearity < 0.001 for both BNP and NT-proBNP concentrations).

### 2.4. Prediction of PH and PVR > 5 Wood Units Using Multivariable Models

Logistic regression models were developed to determine whether natriuretic peptides were independently associated with PH and PVR > 5 Wood units (Table 2). BNP and NT-proBNP were transformed into natural logarithms to fit into logistic regression models. Two models, one including natural logarithm of BNP and one including natural logarithm of NT-proBNP, were developed for prediction of each PH presence and PVR > 5 Wood units (Table 2). For each of developed models, numerous candidacy models showed substantial support (ΔAIC < 2), indicating model selection uncertainty (Supplementary Table S4). Elevated BNP and NT-proBNP concentrations were independently associated with PH and PVR > 5 Wood units. A 2.718-fold increase in BNP or NT-proBNP concentration, corresponding to a one-unit increase on the natural logarithmic scale, was associated with approximately 3.1-fold higher odds of PH for BNP and 2.1-fold higher odds of PH for NT-proBNP. Additionally, females were less likely to have PH, while patients in NYHA III or IV had higher odds of PH presence. A 2.718-fold increase in BNP or NT-proBNP concentration was also associated with approximately 1.7-fold higher odds of PH with a severe precapillary component for both biomarkers. Moreover, older age tended to be associated with higher odds of both PH and PVR > 5 Wood units. In both univariable and multivariable analyses, BNP and NT-proBNP had the highest Wald statistic values for predicting PH, indicating greater predictive value than the other variables included in the models.

### 2.5. Nonlinearity of Association Between Natriuretic Peptides and Hemodynamics

**Figure 4:**
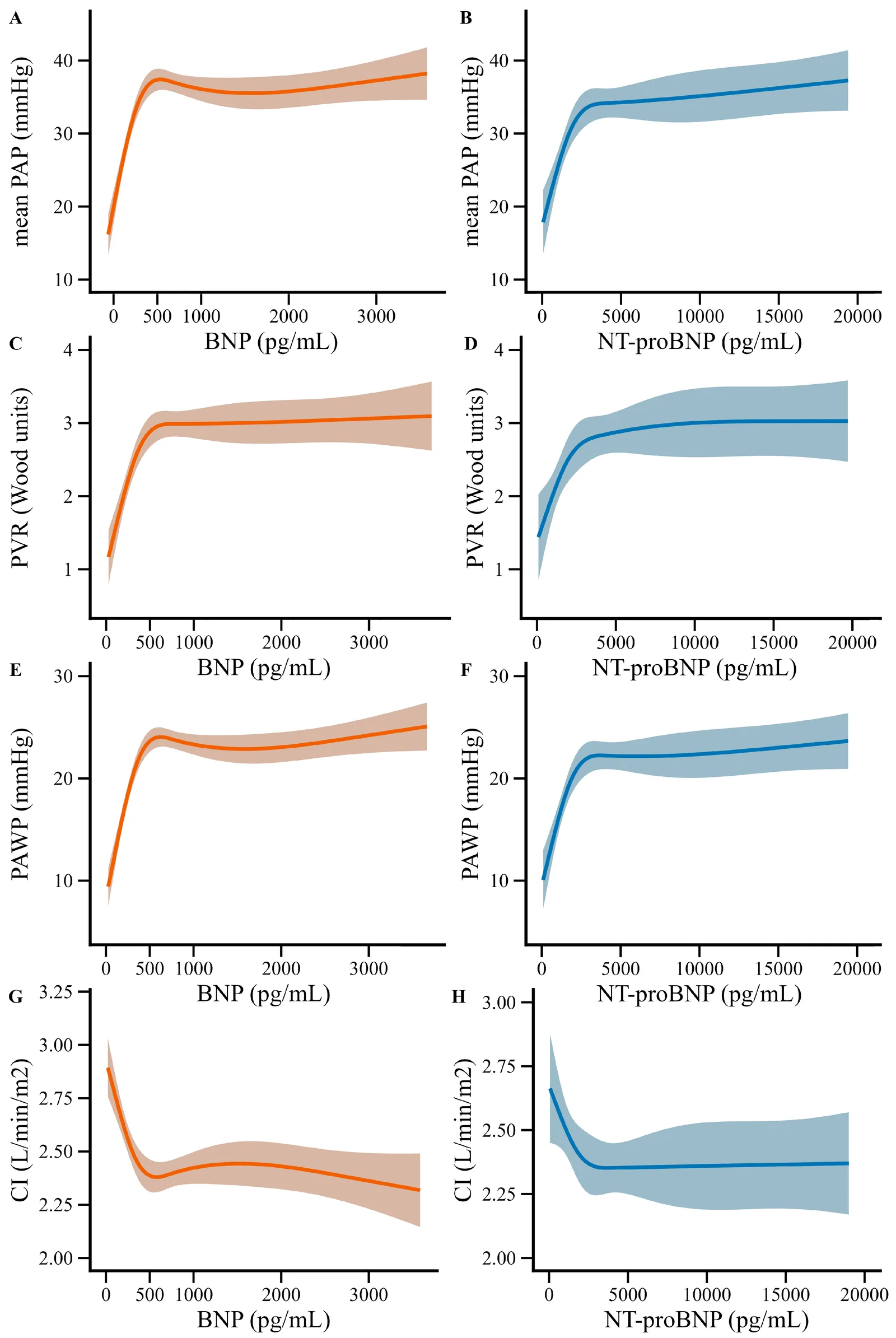
Associations between BNP, NT-proBNP, and mean pulmonary artery pressure (mean PAP) (**A**,**B**), pulmonary vascular resistance (PVR) (**C**,**D**), pulmonary arterial wedge pressure (PAWP) (**E**,**F**), and cardiac index (CI) (**G**,**H**) modeled using restricted cubic splines. The solid line represents the predicted values, and the shaded area indicates 95% confidence intervals. *p*-overall refers to the significance of the overall association, whereas *p*-nonlinearity assesses deviation from linearity. *p*-overall: A < 0.001; B < 0.001; C < 0.001; D < 0.001; E < 0.001; F < 0.001; G < 0.001; H 0.101; *p*-nonlinearity: A < 0.001; B < 0.001; C < 0.001; D 0.001; E < 0.001; F < 0.001; G < 0.001; H 0.075. BNP, B-type natriuretic peptide; CI, cardiac index; mean PAP, mean pulmonary artery pressure; NT-proBNP, N-terminal pro-B-type natriuretic peptide; PAWP, pulmonary arterial wedge pressure; PVR, pulmonary vascular resistance.

Restricted cubic splines were used to further evaluate associations between hemodynamic parameters and natriuretic peptide concentrations beyond the selected cutoff values and their potential nonlinearity (Figure 4). The associations between natriuretic peptides and mean PAP were nonlinear, with mean PAP increasing up to approximately 500 pg/mL for BNP and 3000 pg/mL for NT-proBNP and then plateauing at approximately 35 mmHg (Figure 4). A similar plateau was observed at approximately 500 pg/mL for BNP and 3000 pg/mL for NT-proBNP for PVR, at approximately 3 Wood units, and for PAWP, at approximately 22–23 mmHg. CI decreased with increasing natriuretic peptide concentrations and plateaued at approximately 2.35 L/min/m^2^, again after approximately 500 pg/mL for BNP and 3000 pg/mL for NT-proBNP. An additional figure was generated with the x-axis limited to BNP concentrations up to 1000 pg/mL and NT-proBNP concentrations up to 5000 pg/mL to better visualize changes in hemodynamic parameters before the plateau phase (Supplementary Figure S1).

### 2.6. Discrimination of PH and Selected Hemodynamic Characteristics

**Figure 5:**
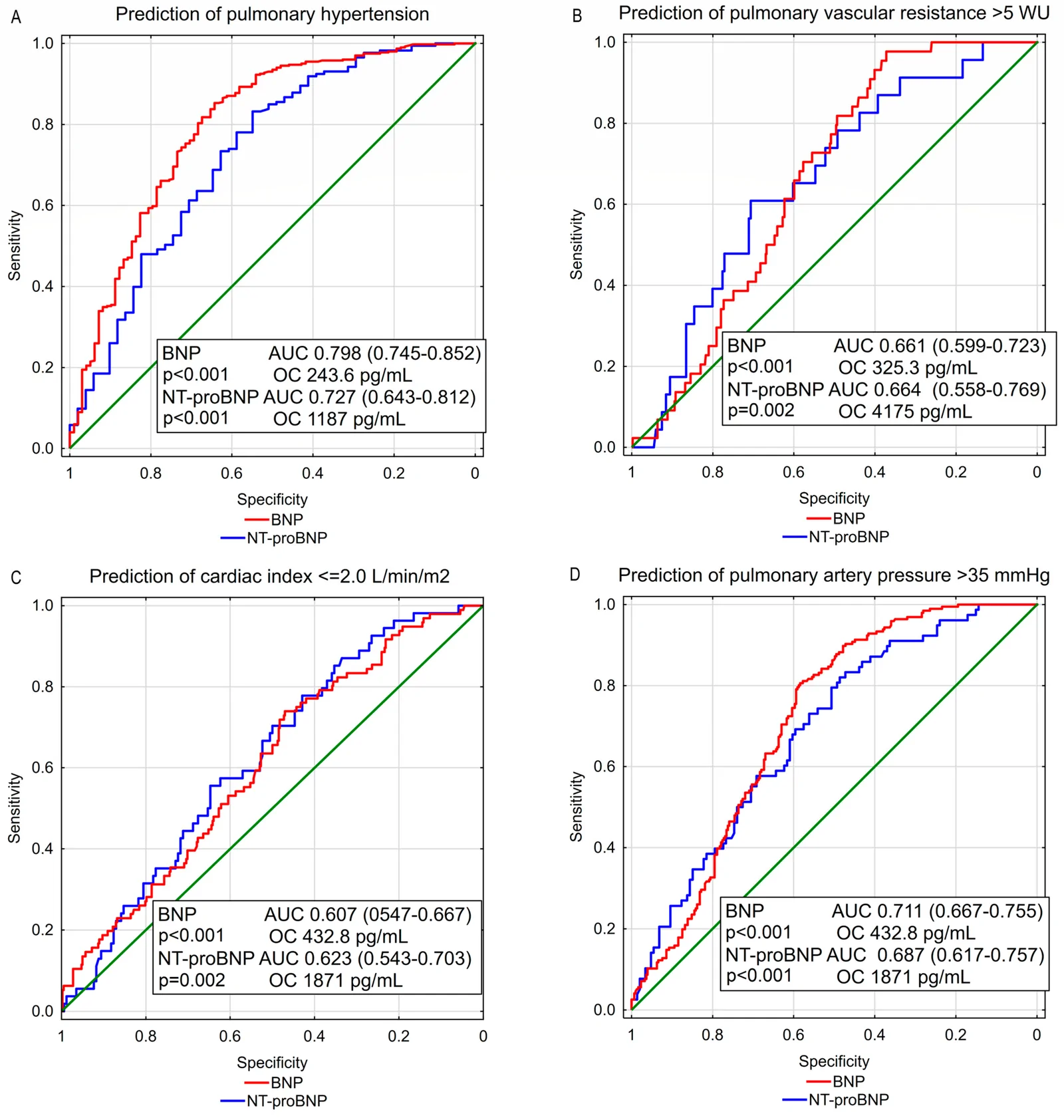
Receiver operating characteristic (ROC) curves picturing the predictive value of BNP and NT-proBNP for pulmonary hypertension (**A**), pulmonary vascular resistance > 5 Wood units (**B**), cardiac index ≤ 2 L/min/m^2^ (**C**), and mean pulmonary artery pressure > 35 mmHg (**D**). *p*-values < 0.05 were considered significant. BNP, B-type natriuretic peptide; CI, cardiac index; NT-proBNP, N-terminal pro-B-type natriuretic peptide; PH, pulmonary hypertension; PVR, pulmonary vascular resistance; ROC, receiver operating characteristic.

The ability of natriuretic peptide concentrations to predict PH was assessed using ROC curves (Figure 5). BNP showed good discrimination for PH, whereas NT-proBNP showed moderate discrimination, with area under the curve (AUC) of 0.798 and 0.727, respectively. Discrimination for PH with a severe precapillary component, defined as PVR > 5 Wood units, was less accurate with AUC 0.661 for BNP and 0.664 for NT-proBNP respectively. Discrimination for low CI, defined as CI ≤ 2.0 L/min/m^2^, was also modest (AUC 0.607 for BNP and AUC 0.623 for NT-proBNP). Discrimination for mean PAP > 35 mmHg was moderate with AUC 0.711 for BNP and AUC 0.687 for NT-proBNP. ROC analysis identified optimal cutoff values for predicting PH using BNP > 243 pg/mL and NT-proBNP > 1187 pg/mL. Detailed analysis of different natriuretic peptides cutoff values for prediction of PH and severe precapillary component (PVR > 5 Wood units) is presented in Supplementary Table S5.

## 3. Discussion

In a relatively large cohort of patients with HFrEF undergoing RHC, circulating natriuretic peptides (BNP and NT-proBNP) were significantly associated with the presence of PH-LHD and with the overall severity of hemodynamic impairment. BNP showed good discrimination for PH (AUC approximately 0.80), while NT-proBNP performed moderately well (AUC approximately 0.72). In contrast, the ability of either biomarker to identify a severe precapillary component—defined in the 2022 European Society of Cardiology/European Respiratory Society (ESC/ERS) PH guidelines as PVR > 5 Wood units—was only modest (AUC approximately 0.66), highlighting an important limitation of natriuretic peptides as stand-alone tools for phenotyping PH-LHD [5].

PH is common in HFrEF and is consistently linked to worse outcomes. Earlier studies demonstrated that elevated pulmonary pressures and right ventricular dysfunction provide independent and additive prognostic information in chronic HF [3,4]. Contemporary PH guidelines emphasize that left heart disease is the leading cause of PH and that RHC remains the reference standard to distinguish hemodynamic phenotypes (isolated postcapillary PH vs combined post- and precapillary) and to quantify PVR, particularly when a significant precapillary component may influence management [5]. In this context, identifying noninvasive markers that could support “triage” to RHC is clinically relevant, especially in advanced HF pathways where invasive evaluation is frequent but still carries risk and limited availability [5].

Natriuretic peptides reflect myocardial wall stress and play a central role in HF diagnosis, risk stratification, and management [2,15]. In HFrEF, higher BNP and NT-proBNP generally signal more advanced disease, higher filling pressures, and greater neurohormonal activation—pathophysiologic processes closely tied to postcapillary PH development [2,5]. The observed stepwise increase in PH prevalence across increasing BNP/NT-proBNP strata in our cohort aligns with the expected biology: as left-sided congestion and overall HF severity increase, pulmonary venous pressures and downstream pulmonary artery pressures rise accordingly [2,5].

Importantly, natriuretic peptides are also widely used in pulmonary arterial hypertension (PAH) and chronic thromboembolic PH as markers of right ventricular strain and prognosis, which strengthens the rationale for evaluating them in PH-LHD—although the underlying mechanisms differ [5,22,23,24,25].

A key finding is that BNP/NT-proBNP discriminated PH presence better than they discriminated PVR > 5 Wood units. This is clinically intuitive. In PH-LHD, natriuretic peptides are predominantly driven by overall HF severity and congestion (left-sided filling pressures), whereas PVR reflects pulmonary vascular remodeling and microvascular disease superimposed on postcapillary loading—processes that may not correlate tightly with acute wall stress at the time of sampling [5]. Therefore, while peptides may “flag” patients likely to have PH, they are less suited to identifying the subgroup with advanced pulmonary vascular disease, which is precisely the group for whom RHC-based hemodynamic phenotyping is most consequential [5].

In PAH, NT-proBNP is deeply embedded in validated multi-parameter risk stratification tools (e.g., COMPERA 2.0, French registry-derived models, REVEAL Lite 2), and low NT-proBNP and BNP values are used as treatment goals and indicators of low risk [5,22,23,24,25]. BNP and NT-proBNP are both sensitive markers for detecting PH; however, BNP may offer greater specificity for distinguishing patients with PH, partly because it is less affected by renal dysfunction, a common comorbidity in this population [26,27,28]. In contrast, NT-proBNP appears to provide stronger prognostic information regarding mortality [29]. However, extrapolating these prognostic models directly to PH-LHD is problematic, because in HFrEF the biomarker integrates left-sided congestion, right ventricular load, renal function, rhythm disorders, and HF therapies [2,5]. Our results support the concept that natriuretic peptides retain diagnostic value in PH-LHD, but they also emphasize that their interpretation must remain phenotype- and context-specific rather than borrowed from PAH algorithms [5,22,23,24,25].

Recent evidence indicates that in hypertensive heart disease, natriuretic peptide concentrations may reflect left ventricular hypertrophy, diastolic dysfunction, and chronic pressure-overload–related wall stress [30,31]. Moreover, in hypertrophic cardiomyopathy, both BNP and NT-proBNP levels were associated with left ventricular thickness and diastolic dysfunction [32,33,34]. In the study of Huang et al., elevated NT-proBNP was associated with left ventricular hypertrophy in patients without HF [35]. Another study showed that left ventricular hypertrophy is associated with elevated NT-proBNP regardless of the presence of arterial (systemic) hypertension [36]. Therefore, in patients with PH and concomitant left ventricular hypertrophy, regardless of etiology, natriuretic peptide concentrations may reflect the combined effects of right ventricular pressure overload and left ventricular structural and functional abnormalities, rather than PH-related right ventricular stress alone.

Importantly, natriuretic peptide concentrations reflect the overall load on the walls of both ventricles [11,37], but in HFrEF their concentrations are significantly influenced by left ventricular wall stress, as left ventricular dimensions reach their highest values across the entire spectrum of HF [38]. As the peptides do not distinguish between the ventricles from which the signal originates, the increase resulting from right ventricular overload in PH is difficult to isolate, which limits diagnostic resolution and explains the overlap in distributions between patients with and without PH [5]. Right heart catheterization enables accurate characterization of these hemodynamic abnormalities, which natriuretic peptides alone are unable to distinguish.

The median NT-proBNP to BNP molar ratio in this study was 2.53 and it was lower in patients with PH compared with patients without PH (molar ratio of NT-proBNP/BNP 2.17 vs. 2.97; *p* < 0.001). This finding may reflect the greater specificity of BNP for distinguishing patients with PH, as suggested by Cavagna et al., who compared BNP and NT-proBNP concentrations in the diagnosis of PAH [27]. NT-proBNP concentrations are more dependent on renal function [39] and consequently BNP may provide more unaffected prediction of ventricular wall stress associated with PH. Nevertheless, NT-proBNP is the most commonly used prognostic parameter in HF [2]. Its dependence on renal function only slightly limits its value as a predictor of poor outcome [40], partly because renal dysfunction itself worsens the prognosis [41], among others, by limiting the use of guideline-directed medical therapy [2]. By comparison, in the study of Suzuki et al., the NT-proBNP to BNP molar ratio was 1.70 in a heterogeneous group of ambulatory patients with at least one cardiovascular risk factor [12]. In this heterogeneous group higher values of the NT-proBNP to BNP molar ratio predicted HF related events [12]. In the mentioned study, patients with HF risk factors, including left ventricular hypertrophy or cardiomyopathies, atrial fibrillation and valve disease, the molar ratio was respectively 2.17; 2.31 and 2.24 [12]. In the study of Wang et al. including hospitalized HF patients, NT-proBNP/BNP molar ratio was 2.37 [14], relatively similar to our study. In recent studies the higher NT-proBNP/BNP predicted cardiovascular events in patients with chronic kidney dysfunction [39] and its elevation preceded worsening of renal function in patients hospitalized with acute HF [13], revealing its potential usefulness as a biomarker for risk stratification of cardiorenal syndrome. The relatively higher NT-proBNP/BNP molar ratio observed in our cohort compared to the study of Suzuki et al. [12] may be explained, at least in part, by the progressive HF phenotype of the enrolled patients, reflecting greater disease severity and a higher burden of ventricular dysfunction. Consistently, a recent study including patients from the PARADIGM-HF trial reported a median NT-proBNP/BNP ratio without molar conversion of 6.25 [42], which closely matched the ratio observed in our cohort (6.20), and showed that this ratio increased with age and renal dysfunction [42]. Nevertheless, results of this study regarding lower NT-proBNP/BNP ratio in PH patients should be interpreted with caution, as measurements of both BNP and NT-proBNP were available in only 160 patients. Furthermore, most patients in the studied cohort had severe systolic dysfunction and progressive HF; therefore, patients without PH did not represent healthy controls, but rather another clinical presentation of severe HF.

Given the performance characteristics shown, BNP/NT-proBNP appear most useful as part of a noninvasive “triage” strategy to identify patients with a higher probability of PH who may benefit from RHC, rather than as biomarkers that could safely replace invasive hemodynamics. This is particularly relevant because ESC/ERS guidelines require invasive measurement of PAWP and PVR to define PH-LHD phenotypes and to confirm a severe precapillary component [5]. In addition, predictive values are influenced by disease prevalence; because the present cohort included patients with advanced HF (and therefore a high PH prevalence), positive predictive values will likely be lower in broader outpatient HFrEF populations [2,5].

Future directions

Future work should validate the proposed cut-offs in less selected HFrEF cohorts and test combined models (natriuretic peptides + echocardiographic parameters + clinical variables) specifically for identifying CpcPH and high PVR states [5]. Longitudinal analyses may be particularly informative, as trajectories or “burden” of natriuretic peptides have shown prognostic relevance in HFrEF populations and could potentially add value beyond single measurements [10,21]. Assessment of atrial natriuretic peptide and of the atrial natriuretic peptide/NT-proBNP ratio may provide an additional tool for predicting the presence of PH and associated hemodynamic abnormalities and represents a promising direction for future studies.

Limitations

The main limitation of this study is its single-center design, which necessitates external validation of the proposed cutoff values in independent populations before routine clinical application. Data on BNP concentration were available in more patients than data on NT-proBNP concentration, therefore the sample size was bigger for BNP than NT-proBNP. The limited sample size of the study may limit the statistical power of subgroup analyses and the generalizability of the findings; therefore, larger multicenter studies are warranted to confirm these results.

## 4. Materials and Methods

### 4.1. Study Sample and Clinical Assessment

This was a retrospective study of patients with HFrEF who underwent RHC. All patients were hospitalized in the Cardiology Department of University Clinical Hospital in Poznan between January 2013 and September 2020 as part of the evaluation for heart transplant. The inclusion criteria were HFrEF, defined as LVEF ≤ 40%, at least 3 months of HF diagnosis, RHC performed during hospitalization, and available BNP or NT-proBNP data. The exclusion criteria were acute clinical status, requiring inotropic support, a diagnosis of precapillary pulmonary hypertension (class I, III, or IV), complex congenital heart disease, pregnancy, and renal replacement therapy. The study analyzed clinical and epidemiologic data, blood tests sampled on admission, including BNP and NT-proBNP, echocardiographic measurements, and RHC parameters.

PH was diagnosed in patients with mean PAP > 20 mmHg according to the 2022 European Society of Cardiology (ESC)/European Respiratory Society (ERS) guidelines [5]. Isolated postcapillary PH (IpcPH) was defined as PVR ≤ 2.0 Wood units and PAWP > 15 mmHg. Combined pre- and postcapillary PH (CpcPH) was diagnosed in patients with PVR > 2.0 Wood units and PAWP > 15 mmHg [5]. A severe precapillary component was defined as PVR > 5 Wood units [5].

For patients with both NT-proBNP and BNP concentrations, NT-proBNP/BNP molar ratios were calculated after conversion of peptide concentrations from mass to molar units, using molecular weights of 8.46 kDa for NT-proBNP and 3.46 kDa for BNP; accordingly, NT-proBNP/BNP molar ratio was defined as NT-proBNP concentration divided by 2.45 times BNP concentration.

### 4.2. Right Heart Catheterization and PH Diagnostic Criteria

RHC was performed using a Swan–Ganz catheter. It was inserted through the vascular access sheath with fluoroscopy guidance. It passed through the superior or inferior vena cava into the right atrium, then through the tricuspid valve into the right ventricle (RV). Furthermore, the catheter was inserted into the pulmonary artery through the pulmonary valve. The pressures—systolic, mean and diastolic—were measured in the atrium, ventricle and pulmonary artery. Then operator wedged the balloon in the branches of the pulmonary artery and measured PAWP. PAWP was used as an estimate of left atrial pressure. After that, the thermodilution method was used to calculate cardiac output (CO). The operator injected 10 mL of 0.9% sodium chloride solution at a temperature of 0 to 5 °C into the right atrium and measured the decrease in temperature in the pulmonary artery. The cardiac output was measured three times and averaged to get the result. It was divided by estimated body surface area to calculate CI. Body surface area was calculated using the Mosteller formula: BSA (m^2^) = √([height (cm) × weight (kg)]/3600) [43].

### 4.3. Statistical Analysis

Statistical analysis was performed using STATISTICA version 13.3 (TIBCO Software Inc., Palo Alto, CA, USA) and R software (version 4.5.2; R Core Team) and RStudio (Posit, Boston, MA, USA). The following R packages were used: glmulti (version 1.0.8), car (version 3.1–5), ggplot2 (version 4.0.3), rms (version 8.1.1), patchwork (version 1.3.2).

Continuous variables are presented as mean (SD) or median (IQR), depending on the presence of a normal distribution. The authors defined a *p*-value of <0.05 as statistically significant. Patients with PH were compared with those without PH. Continuous variables were compared with the Student’s *t* test or Mann–Whitney U test according to the presence of normal distribution and variance homogeneity. Categorical variables were compared with chi-square test or two-sided Fisher’s exact test as appropriate. To assess the relationship of noninvasive findings including natriuretic peptides with invasive RHC parameters Spearman correlations were calculated. The occurrence of PH and PH with severe precapillary component, CI ≤ 2.0 L/min/m^2^, and mean PAP > 35 mmHg was compared between groups of patients stratified by concentrations of BNP (<250; 250–499;500–999; >1000) and NT-proBNP (<1000;1000–1999;2000–3999; >4000). The results were presented as column chart along with *p*-value for Chi2 test to compare prevalence of those characteristics. Post hoc pairwise comparisons of proportions were thereafter calculated with Holm correction for multiple testing. The Cochran–Armitage trend test was calculated to assess presence of increasing trends across BNP and NT-proBNP strata.

To assess for potential nonlinearity, restricted cubic spline curves based on logistic regression models were fitted, showing the association between predicted probability of pulmonary hypertension with each of BNP and NT-proBNP concentrations. Additionally, the potential nonlinear relationship between RHC parameters, namely mean PAP, PVR, PAWP and CI, and BNP as well as NT-proBNP were examined using restricted cubic splines derived from linear regression. Four knots were placed at 5th, 35th, 65th, and 95th percentiles of the distribution of BNP and NT-proBNP concentrations. Predictions with 95% confidence intervals were derived from the fitted models. Nonlinearity was verified using the Likelihood Ratio Test comparing restricted cubic splines versus linear model.

Two logistic regression models for prediction of each PH and PH with PVR > 5 Wood units were developed; one including natural logarithm of BNP and one including natural logarithm of NT-proBNP. Predictors were selected using the best subset selection method based on Akaike information criterion from the following variables: natural logarithm of BNP or NT-proBNP, age, sex, LVEF, NYHA class III or IV. Top-ranked models were selected based on the lowest AIC and presented in the manuscript. Top 5 candidacy models based on Akaike information criterion (AIC) are presented in supplementary data.

Receiver operating characteristic (ROC) curves were generated to assess the ability of BNP and NT-proBNP to predict PH, PH with a severe precapillary component, CI ≤ 2.0 L/min/m^2^, and mean PAP > 35 mmHg. The AUC was calculated for each ROC curve to evaluate discriminative performance; optimal cutoff values of BNP and NT-proBNP concentrations were selected using Youden index method. Sensitivity and specificity were determined for selected cutoff values of BNP (250, 500, and 1000 pg/mL) and NT-proBNP (1000, 2000, and 4000 pg/mL) as well as for optimal cutoff values. Generative AI was used to assist with R-based statistical analysis and superficial text editing; all outputs were reviewed and verified by the authors.

## 5. Conclusions

In summary, BNP and NT-proBNP provide meaningful, readily available information for identifying PH in HFrEF, with BNP showing slightly better discrimination in this dataset. Their ability to detect a severe precapillary component (PVR > 5 Wood units) is limited, supporting their role as screening/triage markers rather than definitive tools for hemodynamic phenotyping. Given the centrality of PVR and PAWP in current PH definitions and management pathways, RHC remains essential when a significant precapillary component is suspected or when clinical decisions depend on precise hemodynamics.

## Figures and Tables

**Table 1:** Baseline characteristics of patients with heart failure with reduced ejection fraction according to pulmonary hypertension presence.

Parameter	All Patients (N = 563)	PH (N = 443)	No PH (N = 120)	*p*-Value
Age (years)	55 (46–60)	55 (47–60)	54 (44–59)	0.229
Females, n (%)	87 (15.5%)	61 (13.8%)	26 (21.7%)	0.034 *
BMI (kg/m^2^)	26.92 (4.34)	27.06 (4.21)	26.37 (4.67)	0.128
Ischemic HF etiology, n (%)	275 (48.8%)	224 (50.6%)	51 (42.5%)	0.117
Heart rate during RHC (beats/min)	70 (64–79)	71 (65–80)	66 (60–75)	<0.001 *
LVEF (%)	20 (15–25)	20 (15–25)	25 (20–26.5)	<0.001 *
NYHA class I	4 (0.7%)	3 (0.7%)	1 (0.8%)	<0.001 *
NYHA class II	169 (30.0%)	113 (25.5%)	56 (46.6%)	
NYHA class III	309 (54.9%)	252 (56.9%)	57 (47.5%)	
NYHA class IV	81 (14.4%)	75 (16.9%)	6 (5.0%)	
NYHA class III or IV	390 (69.3%)	327 (73.8%)	63 (52.5%)	<0.001 *
Comorbidities				
Diabetes mellitus, n (%)	135 (24.0%)	113 (25.6%)	22 (18.3%)	0.102
Kidney dysfunction (GFR <60 mL/min/1.73 m^2^)	199 (35.4%)	167 (37.7%)	32 (26.7%)	0.025 *
Chronic obstructive pulmonary disease, n (%)	44 (7.8%)	32 (7.2%)	12 (10%)	0.312
Atrial fibrillation at hospitalization, n (%)	110 (19.5%)	91 (20.5%)	19 (15.8%)	0.248
History of paroxysmal AF, n (%)	89 (15.8%)	71 (16.0%)	18 (15.0%)	0.784
Systemic arterial hypertension, n (%)	195 (34.6%)	156 (35.2%)	39 (32.5%)	0.579
Laboratory parameters				
TSH (μIU/mL)	1.69 (0.97–2.87)	1.80 (1.02–3.02)	1.36 (0.79–2.40)	0.002 *
BNP (pg/mL)	503.8 (249.6–976.9)	598.4 (323.0–1119.2)	163.7 (83.2–408.7)	<0.001 *
NT-proBNP (pg/mL)	2381.5 (1165–4888.5)	2729 (1595–5493)	1089 (461–2632)	<0.001 *
Molar ratio of NT-proBNP/BNP	2.53 (1.40–2.88)	2.17 (1.30–2.62)	2.97 (2.27–3.79)	<0.001 *
Ratio of NT-proBNP/BNP	6.20 (3.43–7.05)	4.79 (3.20–6.43)	7.28 (5.56–9.29)	<0.001 *
GFR by MDRD (mL/min/1.73 m^2^)	69.8 (22.8)	68.0 (21.7)	76.1 (25.4)	<0.001 *
Hemoglobin (mmol/L)	8.91 (0.96)	8.88 (0.97)	9.01 (0.93)	0.171
RBC (10^6^/μL)	4.74 (0.57)	4.73 (0.54)	4.77 (0.67)	0.557

* *p*-values < 0.05 were considered significant. Data are presented as mean (SD), median (IQR), or n (%), as appropriate. *p*-values refer to comparisons between patients with and without pulmonary hypertension. AF, atrial fibrillation; BMI, body mass index; GFR, glomerular filtration rate; HFrEF, heart failure with reduced ejection fraction; IQR, interquartile range; MDRD, Modification of Diet in Renal Disease; NYHA, New York Heart Association; PH, pulmonary hypertension; RBC, red blood cell count; SD, standard deviation; TSH, thyroid-stimulating hormone.

**Table 2:** Univariable and multivariable logistic regression models for predicting pulmonary hypertension and pulmonary vascular resistance > 5 Wood units.

Predictor	Univariable OR (95% CI) for Predicting PH	Wald Statistic	*p*-Value	Univariable OR (95% CI) for Predicting PVR > 5 Wood Units	Wald Statistic	*p*-Value	
age (years)	1.010 (0.991–1.030)	1.083	0.298	1.024 (0.993–1.055)	2.350	0.128	
LVEF (%)	0.945 (0.919–0.973)	14.807	<0.001 *	0.984 (0.944–1.026)	0.606	0.456	
female sex	0.577 (0.346–0.963)	4.431	0.035 *	1.103 (0.518–2.346)	0.067	0.800	
NYHA III, IV class	2.550 (1.682–3.867)	19.439	<0.001 *	2.766 (1.276–5.995)	6.610	0.010 *	
Ln(BNP)	3.122 (2.402–4.058)	72.450	<0.001 *	1.704 (1.235–2.351)	10.604	0.001 *	
Ln(NT-proBNP)	2.205 (1.619–3.003)	25.165	<0.001 *	1.620 (1.070–2.453)	5.190	0.023 *	
Multivariable models predicting PH							
Model I (BNP) R^2^ = 0.324	OR (95% CI)	Wald statistic	*p*-value	Model II (NT-proBNP) R^2^ = 0.244	OR (95% CI)	Wald statistic	*p*-value
Ln(BNP)	3.081 (2.361–4.020)	68.651	<0.001 *	Ln(NT-proBNP)	2.104 (1.515–2.923)	19.688	<0.001 *
female sex	0.443 (0.236–0.832)	6.400	0.011 *	Age (years)	1.031 (0.998–1.064)	3.434	0.064
NYHA III or IV	1.663 (0.980–2.822)	3.553	0.059	NYHA III or IV	1.746 (0.856–3.560)	2.352	0.125
Multivariable models predicting PVR > 5 Wood units							
Model I (BNP) R^2^ = 0.065	OR (95% CI)	Wald statistic	*p*-value	Model II (NT-proBNP) R^2^ = 0.072	OR (95% CI)	Wald statistic	*p*-value
Ln(BNP)	1.780 (1.278–2.478)	11.683	<0.001 *	Ln(NT-proBNP)	1.714 (1.101–2.669)	5.685	0.017 *
Age (years)	1.029 (0.995–1.064)	2.815	0.093	Age (years)	1.032 (0.988–1.077)	1.981	0.159

* *p*-values <0.05 were considered significant. ORs are presented with 95% confidence intervals. *p*-values are shown for each predictor. BNP and NT-proBNP were natural log-transformed before inclusion in the models. OR, odds ratio. In this table, CI denotes confidence interval.

## Data Availability

The datasets used and/or analyzed during the current study are available from the corresponding author on reasonable request.

## Supplementary Materials

The following supporting information can be downloaded at: https://www.mdpi.com/article/10.3390/ijms27177776/s1.

## Abbreviations

The following abbreviations are used in this manuscript:

AUC	Area under the curve
BNP	B-type natriuretic peptide
BSA	Body surface area
CI	Cardiac index (except in Table 2, where CI denotes confidence interval)
CO	Cardiac output
COPD	Chronic obstructive pulmonary disease
CpcPH	Combined pre- and postcapillary pulmonary hypertension
ESC	European Society of Cardiology
ERS	European Respiratory Society
GDPR	General Data Protection Regulation
GFR	Glomerular filtration rate
HF	Heart failure
HFrEF	Heart failure with reduced ejection fraction
IpcPH	Isolated postcapillary pulmonary hypertension
IQR	Interquartile range
LHD	Left heart disease
LVEF	Left ventricular ejection fraction
MDRD	Modification of Diet in Renal Disease
NT-proBNP	N-terminal pro-B-type natriuretic peptide
NYHA	New York Heart Association
OR	Odds ratio
PAH	Pulmonary arterial hypertension
PAP	Pulmonary artery pressure
PAWP	Pulmonary arterial wedge pressure
PH	Pulmonary hypertension
PH-LHD	Pulmonary hypertension due to left heart disease
PVR	Pulmonary vascular resistance
RBC	Red blood cell count
RHC	Right heart catheterization
ROC	Receiver operating characteristic
RV	Right ventricle
RVSP	Right ventricular systolic pressure
SD	Standard deviation
TSH	Thyroid-stimulating hormone
