# Supplementary material for: Natriuretic Peptides as Predictors for the Diagnosis of Pulmonary Hypertension Secondary to Left Heart Disease and for the Assessment of Its Severity
Source: Int J Mol Sci. 2026 Aug 30;27(17):7776. doi: 10.3390/ijms27177776 (PMC13566357; doi:10.3390/ijms27177776)
Supplement: Supplementary file 1 [file ijms-27-07776-s001.zip › ijms-4471547-supplementary.pdf]

Supplementary Table S1. Echocardiographic and right heart catheterization parameters according to pulmonary hypertension presence.

Variable	Value (All patients, N=563)	Value in Patients with PH (N=443)	Value in Patients without PH (N=120)	p-value
Echocardiographic parameters				
LVEF [%]	20 (15-25)	20 (15-25)	25 (20-26.5)	<0.001
LV diameter [mm]	72.5 (11.3)	73.1 (11.0)	70.6 (12.1)	0.036*
RV diameter [mm]	36 (31-40)	36 (32-41)	33 (30-37)	<0.001*
LAD [mm]	53 (48-58)	54 (49-59)	49 (43-54)	<0.001*
IVS [mm]	10 (9-11)	9 (9-10)	10 (9-11)	0.005*
PW [mm]	10 (9-10)	9 (9-10)	10 (9-11)	0.022*
RVSP [mmHg]	42 (34-51)	45 (36-55)	34 (30-38)	<0.001*
Moderate or severe TR	254 (48.0%)	189 (44.4%)	21 (18.1%)	<0.001*
Severe TR	53 (9.8%)	48 (11.3%)	5 (4.3%)	0.041*
Moderate MR	345 (63.6%)	291 (68.3%)	54 (46.6%)	<0.001*
Severe MR	66 (12.3%)	62 (14.6%)	4 (3.5%)	0.002*
Right heart catheterization parameters				
Mean PAP [mmHg]	32.16 (12.74)	36.55 (10.63)	15.97 (3.24)	<0.001*
Mean RAP [mmHg]	8 (4-12)	9 (5-14)	3 (2-6)	<0.001*
AoPs [mmHg]	112.5 (101-124)	112 (101-123)	114 (103-125)	0.352
AoPm [mmHg]	90 (82-97)	90 (82-97)	89.5 (79.5-97)	0.547
RVPs [mmHg]	43 (31-56)	48 (38-60)	25 (21-28)	<0.001*
RVPd [mmHg]	5.5 (2-10)	7 (3-10)	1 (1-4)	<0.001*
RVPed [mmHg]	12 (8-20)	15 (10-20)	7 (4-11)	<0.001*
CO [L/min]	4.785 (4.01-5.5)	4.67 (4.00-5.47)	4.98 (4.20-5.67)	0.075
CI [L/min/m²]	2.47 (2.08-2.79)	2.40 (2.07-2.76)	2.58 (2.19-2.99)	0.009*
PAWP [mmHg]	21 (14-27)	24 (19-28)	9 (7-11)	<0.001*
PVR [WU]	2.10 (1.47-3.40)	2.48 (1.68-3.80)	1.44 (1.06-1.89)	<0.001*
PVR [dyn·s·cm⁻⁵]	170 (118-273)	198.5 (134.0-305.0)	114.9 (84.6-150.9)	<0.001*
TPG [mmHg]	10 (7-15)	12 (8-17)	7 (5-8.5)	<0.001*

* p values < 0.05 were considered statistically significant

Abbreviations: ACEI, angiotensin-converting enzyme inhibitor; AF, atrial fibrillation; AoPm, mean aortic pressure; AoPs, systolic aortic pressure; ARB, angiotensin receptor blocker; ARNI, angiotensin receptor-neprilysin inhibitor; BNP, B-type natriuretic peptide; CI, cardiac index; CO, cardiac output; GFR MDRD, glomerular filtration rate estimated using the Modification of Diet in Renal Disease formula; IHD, ischemic heart disease; IVS, interventricular septum; LAD, left atrial diameter; LV, left ventricle; LVEF, left ventricular ejection fraction; MR, mitral regurgitation; NYHA, New York Heart Association; PAP, pulmonary artery pressure; PAWP, pulmonary artery wedge pressure; PH, pulmonary hypertension; PW, posterior wall; PVR, pulmonary vascular resistance; RAP, right atrial pressure; RHC, right heart catheterization; RV, right ventricle; RVSP, right ventricular systolic pressure; RVPd, right ventricular diastolic pressure; RVPed, right ventricular end-diastolic pressure; RVPs, right ventricular systolic pressure; TPG, transpulmonary gradient; TR, tricuspid regurgitation.

Supplementary Table S2. Correlations between invasive right heart catheterization findings and non-invasive parameters.

Variable	PH	HR	Mean PAP	Mean RAP	AoPs	AoPm	RVPs	RVPd	RVPed	CO	CI	PAWP	PVR	TPG
age	.051	-.053	.086*	.002	.105*	.097*	.122*	.050	.041	-.103*	-.090*	.062	.153**	.015
Male sex	.089*	.077	.091*	.135*	-.047	.016	.017	.021	.114*	.254**	.023	.083*	-.106*	.078
IHD etiology	.066	-.129*	.122*	-.079	.077	.046	.192**	-.053	-.011	.063	.030	.042	.132*	.083
DM	.069	.043	.151**	.099*	.159**	.165**	.161**	.098*	.077	.033	-.020	.135*	.094*	.143*
COPD	-.027	.034	.069	.064	.014	.007	.056	.104*	.095*	-.008	-.001	.045	.086*	.110*
Paroxysmal AF	.012	-.046	.077	-.011	-.019	-.025	.071	.046	.052	-.038	-.098*	.072	.059	.061
AF in ECG	.049	.223**	.029	.093*	.038	.072	.012	-.040	-.012	.027	-.054	.038	.008	.012
arterial hypertension	.023	.008	.026	-.027	.244**	.220**	.004	-.020	-.007	.030	-.067	-.005	.050	.082
NYHA III/IV	.185**	.130**	.209**	.230**	-.172**	-.119*	.210**	.188**	.197**	-.115*	-.103*	.230**	.176**	-.001
height (m)	-.009	.072	-.004	.000	-.097*	-.089*	.012	-.113*	-.175**	.047	-.202**	-.021	-.003	.018
BMI	.075	.100*	.108*	.065	.287**	.264**	.100*	.019	.058	.333**	.046	.073	-.066	.065
TSH uIU/mL	.136*	.011	.152**	.165**	-.203**	-.192**	.140*	.148*	.086*	-.114*	-.056	.131*	.126*	.079
BNP	.423**	.161**	.458**	.346**	-.320**	-.229**	.424**	.304**	.268**	-.272**	-.215**	.446**	.385**	.107*
NT-proBNP	.324**	.270**	.425**	.339**	-.458**	-.380**	.372**	.326**	.249**	-.154*	-.136*	.426**	.307**	.110
GFR MDRD	-.156**	.011	-.184**	-.154**	.094*	.058	-.194**	-.179**	-.131*	.106*	.135*	-.161**	-.198**	.183**
Hgb	-.102*	-.075	-.110*	-.068	.152**	.194**	-.139*	-.094*	-.060	-.031	-.116*	-.139**	-.040	.068
RBC	-.018	-.011	-.016	.022	.105*	.167**	-.016	.001	-.004	-.047	-.156**	-.036	.012	.083*
LVEF	-.161**	-.109*	-.110*	-.081	.214**	.167**	-.104*	-.142*	-.085*	.133*	.141**	-.151**	-.072	.009
LV diameter [mm]	.092*	-.016	.100*	.034	-.076	-.044	.087	.036	.032	.166**	.042	.149**	-.080	-.039
RV diameter [mm]	.278**	.197**	.354**	.234**	-.074	-.019	.323**	.156**	.219**	.118*	-.014	.370**	.119*	.053
LAD [mm]	.218**	.184**	.284**	.305**	-.139*	-.055	.261**	.211**	.203**	.082	-.017	.261**	.147**	.140**
RVSP [mmHg]	.417**	.152*	.598**	.314**	-.108*	-.049	.621**	.279**	.328**	-.159*	-.135*	.519**	.487**	.211**

Correlation heatmap legend						
≤ −0.40	−0.20	−0.05	0	0.05	0.20	≥ 0.40

Values are correlation coefficients. Blue indicates negative correlations, white indicates no or negligible correlation, and red indicates positive correlations. * $p < 0.05$; ** $p < 0.001$; p values < 0.05 were considered statistically significant

Abbreviations: COPD, chronic obstructive pulmonary disease; DM, diabetes mellitus; ECG, electrocardiogram; Hgb, hemoglobin; HR, heart rate; NT-proBNP, N-terminal pro-B-type natriuretic peptide; RBC, red blood cell count; TSH, thyroid-stimulating hormone.

Supplementary Table S3. Patient characteristics according to BNP concentration categories.

Variable	BNP<250 pg/mL (N=126)	250 pg/mL ≤ BNP <500 pg/mL (N=122)	500 pg/mL ≤ BNP <1000 pg/mL (N=133)	BNP ≥ 1000 pg/mL (N=118)	p-value
Pulmonary hypertension	63 (50%)	105 (86.1%)	122 (91.7%)	111 (94.1%)	<0.001
Age [years]	55 (47-60)	57.5 (48-60)	54 (48-60)	53 (40-59)	0.023
Female sex	21 (16.7%)	24 (19.7%)	14 (10.5%)	20 (17.0%)	0.227
BMI [kg/m²]	27.66 (3.81)	27.69 (4.02)	27.38 (4.05)	24.37 (4.13)	<0.001*
IHD etiology	70 (55.6%)	66 (54.1%)	66 (49.6%)	45 (38.1%)	0.030*

NYHA class III/IV	79 (62.7%)	80 (65.6%)	94 (70.7%)	97 (82.2%)	0.005*
Persistent AF	27 (21.4%)	45 (36.9%)	37 (27.8%)	20 (17.0%)	0.003*
History of paroxysmal AF**	22 (17.5%)	14 (11.5%)	18 (13.5%)	7 (5.9%)	0.049*
GFR MDRD	74.7 (23.9)	69.0 (23.4)	69.1 (19.5)	64.6 (21.4)	0.005*
LVEF [%]	25 (17-30)	20 (15-25)	20 (15-25)	15 (15-20)	<0.001*
Moderate TR	18 (14.3%)	54 (44.3%)	53 (39.8%)	63 (53.4%)	<0.001*
Severe TR	2 (1.6%)	7 (5.7%)	15 (11.3%)	23 (19.5%)	<0.001*
Moderate MR	60 (47.6%)	75 (61.5%)	87 (65.4%)	84 (71.2%)	0.001*
Severe MR	5 (4.0%)	17 (13.9%)	14 (10.5%)	22 (18.6%)	0.003*
Mean PAP [mmHg]	22.87 (9.71)	33.78 (11.93)	37.15 (12.74)	36.39 (10.31)	<0.001*
Mean RAP [mmHg]	5 (3-8)	7 (4-10)	9 (4.5-14)	10 (6-15)	<0.001*
AoPs [mmHg]	117 (108-130)	115 (105-129)	111.5 (99-119)	105 (96-115)	<0.001*
RVPed [mmHg]	9 (6-15)	13 (8-20)	15 (10-25)	15 (10-20)	<0.001*
CO [L/min]	5.15 (4.57-5.90)	4.93 (4.17-5.50)	4.50 (3.95-5.40)	4.50 (3.60-5.33)	<0.001*
CI [L/min/m²]	2.60 (2.35-3.01)	2.49 (2.15-2.84)	2.30 (2.04-2.69)	2.34 (2.0-2.70)	<0.001*
PAWP [mmHg]	13 (9-17)	22 (16-27)	24 (19-28)	24 (21-28)	<0.001*
PVR [WU]	1.5 (1.1-2.2)	2.07 (1.46-3.39)	2.64 (1.84-3.86)	2.61 (1.75-3.89)	<0.001*
PVR [dyn·s·cm^{-5}]	120 (88-176)	166 (117-271)	212 (147-308.5)	214 (140-311)	<0.001*
TPG [mmHg]	8 (6-10)	10 (7-14)	12 (8-17)	12 (8-17)	<0.001*
ARNI	3 (2.4%)	6 (4.9%)	10 (7.5%)	6 (5.1%)	0.300
ACEI/ARB**	120 (95.2%)	102 (83.6%)	105 (78.9%)	74 (62.7%)	0.369

* p values <0.05 were considered significant

**without ARNI

Supplementary Figure S1. Associations between BNP, NT-proBNP, and mean pulmonary artery pressure (A,B), pulmonary vascular resistance (PVR) (C,D), pulmonary arterial wedge pressure (PAWP) (E,F), and cardiac index (CI) (G,H), modeled using restricted cubic splines. The x-axis was restricted to BNP concentrations up to 1000 pg/mL and NT-proBNP concentrations up to 5000 pg/mL. The solid line represents the predicted values, and the shaded area indicates 95% confidence intervals. * p values <0.05 were considered significant

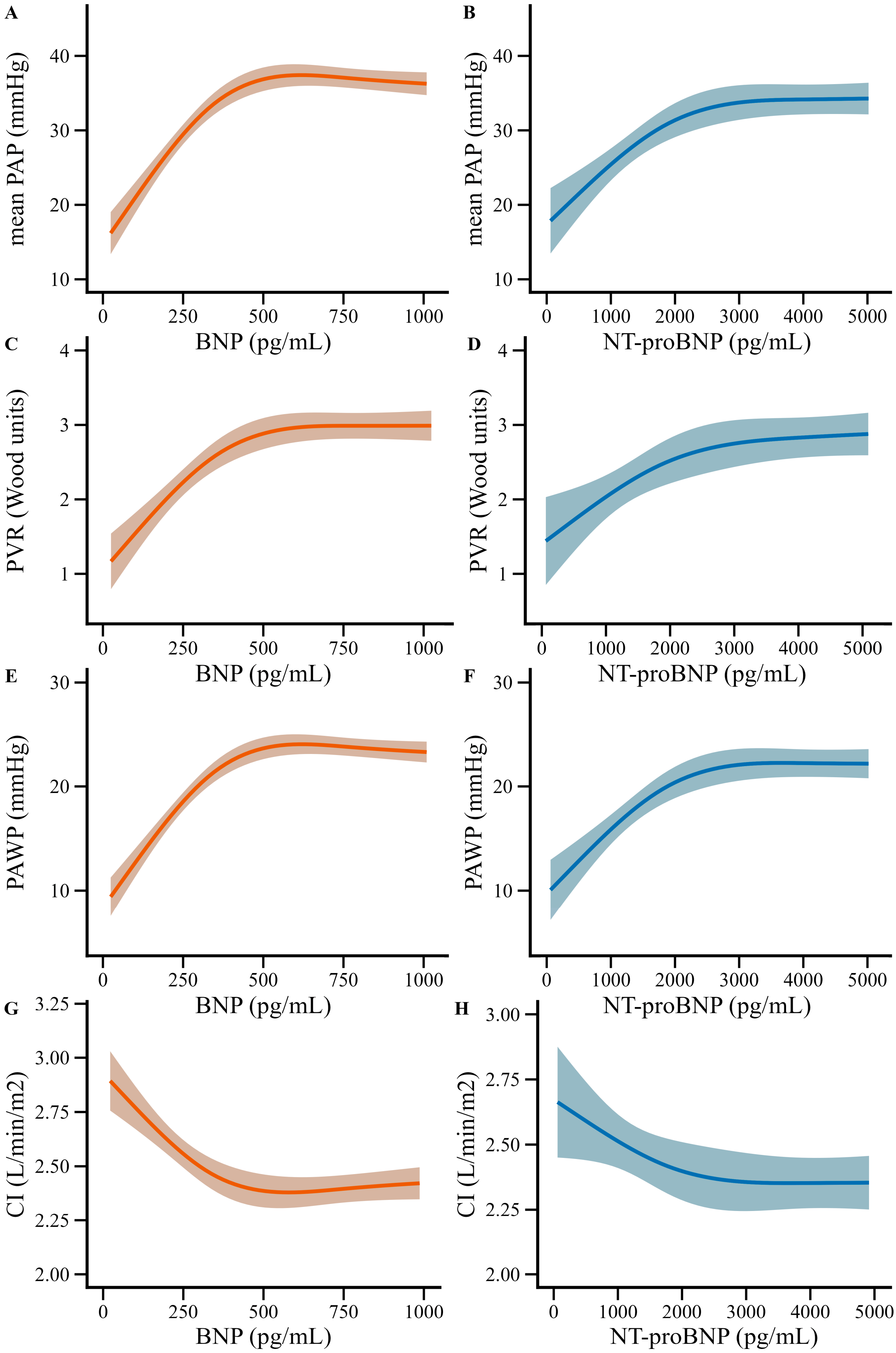
A
mean PAP (mmHg)
10
20
30
40
0
250
500
750
1000
BNP (pg/mL)
B
mean PAP (mmHg)
10
20
30
40
0
1000
2000
3000
4000
5000
NT-proBNP (pg/mL)
C
PVR (Wood units)
1
2
3
4
0
250
500
750
1000
BNP (pg/mL)
D
PVR (Wood units)
1
2
3
4
0
1000
2000
3000
4000
5000
NT-proBNP (pg/mL)
E
PAWP (mmHg)
10
20
30
0
250
500
750
1000
BNP (pg/mL)
F
PAWP (mmHg)
10
20
30
0
1000
2000
3000
4000
5000
NT-proBNP (pg/mL)
G
CI (L/min/m2)
2.00
2.25
2.50
2.75
3.00
3.25
0
250
500
750
1000
BNP (pg/mL)
H
CI (L/min/m2)
2.00
2.25
2.50
2.75
3.00
0
1000
2000
3000
4000
5000
NT-proBNP (pg/mL)

Supplementary Table S4. Top five candidate models ranked by Akaike information criterion (AIC) for each multivariable model predicting pulmonary hypertension (PH) or PH with pulmonary vascular resistance (PVR) > 5 Wood units. Several candidacy models demonstrated substantial support (ΔAIC < 2), indicating uncertainty in model selection.

**Model**	**Predictors of PH (using Ln(BNP))**	**k**	**AIC**	**ΔAIC**
M1	Ln(BNP) + female sex + NYHA class III or IV	3	385.667	0.000
M2	Ln(BNP) + female sex + NYHA class III or IV + LVEF (%)	4	386.834	1.168
M3	Ln(BNP) + female sex	2	387.165	1.498
M4	Ln(BNP) + female sex + NYHA class III or IV + age (years)	4	387.475	1.808
M5	Ln(BNP) + female sex + age (years)	3	388.463	2.796
**Model**	**Predictors of PH (using Ln(NT-proBNP))**	**k**	**AIC**	**ΔAIC**
M1	Ln(NT-proBNP) + age (years)+ NYHA class III or IV	3	209.133	0.000
M2	Ln(NT-proBNP) + age (years)	2	209.461	0.328
M3	Ln(NT-proBNP) + NYHA class III or IV	2	210.501	1.368
M4	Ln(NT-proBNP) + age (years) + NYHA class III or IV + female sex	4	210.843	1.710
M5	Ln(NT-proBNP) + age (years) + NYHA class III or IV + LVEF (%)	4	211.107	1.974
Model	Predictors of PVR >5 Wood units (using Ln(BNP))	k	AIC	ΔAIC
M1	Ln(BNP) + age (years)	2	288.476	0.000
M2	Ln(BNP) + age (years) + NYHA class III or IV	3	288.645	0.169
M3	Ln(BNP) + NYHA class III or IV	2	288.751	0.275
M4	Ln(BNP)	1	289.493	1.017
M5	Ln(BNP) + age (years) + LVEF (%)	3	289.979	1.504
**Model**	**Predictors of PVR >5 Wood units (using Ln(NT-proBNP))**	**k**	**AIC**	**ΔAIC**
M1	Ln(NT-proBNP) + age (years)	2	146.314	0.000
M2	Ln(NT-proBNP)	1	146.476	0.163
M3	Ln(NT-proBNP) + female sex	2	146.703	0.389

M4	Ln(NT-proBNP) + age (years) + female sex	3	146.851	0.537
M5	Ln(NT-proBNP) + female sex + LVEF (%)	3	147.450	1.136

ΔAIC was calculated relative to the model with the lowest AIC value.

k, number of predictors included in the model. Ln(BNP), natural logarithm of B-type natriuretic peptide ; NYHA, New York Heart Association; LVEF, left ventricular ejection fraction;

Supplementary Table S5. Sensitivity, specificity, positive predictive value, negative predictive value, and accuracy of selected natriuretic peptide cut-off values for predicting pulmonary hypertension and pulmonary vascular resistance >5 Wood units.

BNP for prediction of pulmonary hypertension (AUC=0.798)						
BNP cut-off value	125 pg/mL	OC - 243.6 pg/mL	250 pg/mL	500 pg/mL	1000 pg/mL	2000 pg/mL
Sensitivity	0.955	0.853	0.845	0.581	0.279	0.097
Specificity	0.398	0.643	0.643	0.806	0.929	0.969
PPV	0.867	0.907	0.906	0.925	0.941	0.929
NPV	0.684	0.516	0.504	0.32	0.239	0.208
Accuracy	0.846	0.812	0.806	0.625	0.407	0.269
NT-proBNP for prediction of pulmonary hypertension (AUC=0.727)						
NT-proBNP cut-off value	500 pg/mL	1000 pg/mL	OC - 1187 pg/mL	2000 pg/mL	4000 pg/mL	10000 pg/mL
Sensitivity	0.977	0.873	0.832	0.653	0.393	0.104
Specificity	0.255	0.451	0.549	0.647	0.843	0.961
PPV	0.816	0.844	0.862	0.863	0.895	0.900
NPV	0.765	0.511	0.491	0.355	0.291	0.240
Accuracy	0.813	0.777	0.768	0.652	0.496	0.299
BNP for prediction of pulmonary vascular resistance >5 Wood units (AUC=0.661)						
BNP cut-off value	125 pg/mL	250 pg/mL	OC - 325.3 pg/mL	500 pg/mL	1000 pg/mL	2000 pg/mL
Sensitivity	1.000	0.977	0.977	0.727	0.364	0.068
Specificity	0.126	0.271	0.372	0.515	0.773	0.914
PPV	0.1	0.115	0.131	0.127	0.134	0.071
NPV	1	0.992	0.994	0.951	0.926	0.910
Accuracy	0.203	0.333	0.426	0.534	0.737	0.839
NT-proBNP for prediction of pulmonary vascular resistance >5 Wood units (AUC=0.664)						
NT-proBNP cut-off value	500 pg/mL	1000 pg/mL	2000 pg/mL	4000 pg/mL	OC - 4175 pg/mL	10000 pg/mL
Sensitivity	1.000	0.913	0.783	0.609	0.609	0.130

Specificity	0.085	0.214	0.438	0.692	0.706	0.915
PPV	0.111	0.117	0.137	0.184	0.192	0.150
NPV	1.000	0.956	0.946	0.939	0.940	0.902
Accuracy	0.179	0.286	0.473	0.683	0.696	0.835

Abbreviations: AUC, area under the curve; NPV, negative predictive value; OC, optimal cut-off; PPV, positive predictive value.
